# Quantitative assessment of alkali and carbon nanotube reinforcement effects on the tensile reliability of sustainable sisal fiber bio-based epoxy composites

**DOI:** 10.1038/s41598-026-42131-9

**Published:** 2026-03-06

**Authors:** Kishor Joshi, Pavan Hiremath, Shivashankarayya Hiremath, D. V. Ghewade, H. M. Vishwanatha, Kiran Keshyagol

**Affiliations:** 1 Manipal Institute of Technology, Manipal Academy of Higher Education, Manipal, India; 2 Department of Mechanical and Mechatronics Engineering, Dr. A. D. Shinde College of Engineering, Bhadgaon, Maharashtra 416502 India

**Keywords:** Sisal fiber composites, Bio-based epoxy, Multi-walled carbon nanotubes, Sustainable composites, SDG 9, SDG 12, SDG 13, Engineering, Materials science

## Abstract

The present study investigates a two-stage reinforcement strategy to enhance the tensile performance and reliability of sisal fiber–reinforced bio-based epoxy composites, aligning material development with sustainability-driven design principles. In the first stage, sisal fiber mats were treated with 4 wt% and 5 wt% NaOH to improve fiber–matrix interfacial efficiency, while in the second stage, multi-walled carbon nanotubes (MWCNTs) were incorporated into the epoxy matrix at low weight fractions of 0.15, 0.25, and 0.35 wt% using a combined mechanical stirring and ultrasonication approach. Tensile testing conducted in accordance with ASTM D3039 revealed a systematic increase in ultimate tensile strength (UTS) from 71.24 MPa for untreated composites to 103.32 MPa for 5 wt% NaOH-treated composites, corresponding to an improvement of approximately 45% due to enhanced interfacial bonding. Subsequent CNT modification further improved tensile performance, with an optimum response observed at 0.25 wt% MWCNT, achieving a maximum UTS of 129.36 MPa and an elastic modulus of 8.1 GPa. Regression-based mathematical modelling captured the near-linear strengthening behavior induced by alkali treatment and the non-linear saturation-dominated response associated with CNT addition, with model predictions remaining within experimental scatter. Statistical reliability assessment using Weibull analysis demonstrated reduced strength variability for alkali-treated and optimally CNT-modified composites. Fracture surface analysis using scanning electron microscopy revealed a clear transition from interfacial debonding and fiber pull-out to cohesive fracture, crack bridging, and crack deflection mechanisms at optimized reinforcement levels. This study quantifies the combined effect of alkali treatment and low-loading CNTs on sisal bio-epoxy tensile behavior, achieving ~ 82% strength improvement with an optimum at 0.25 wt% CNT, while enhancing stiffness and maintaining controlled variability within the tested range. By integrating renewable natural fibers, low nanofiller content, and data-driven modelling, this study contributes to sustainable materials innovation (SDG 9), responsible material utilization (SDG 12), and reduced environmental impact through lightweight composite design (SDG 13).

## Introduction

The growing demand for lightweight, energy-efficient, and environmentally responsible structural materials has accelerated the development of natural fiber-reinforced polymer composites for semi-structural and moderately load-bearing applications^1,2^. Among various lignocellulosic reinforcements, sisal fiber has received considerable attention due to its low density, favorable specific strength, renewability, and widespread availability^3,4^. However, despite these advantages, the practical use of sisal fiber-based composites remains constrained by intrinsic limitations associated with weak fiber–matrix interfacial adhesion, moisture sensitivity, and variability in mechanical performance. The hydrophilic nature of sisal fibers, arising from the presence of hydroxyl-rich cellulose and hemicellulose constituents, leads to poor compatibility with typically hydrophobic polymer matrices^5,6^. This interfacial mismatch restricts effective stress transfer under mechanical loading, resulting in premature fiber pull-out, interfacial debonding, and reduced composite strength. Surface modification of natural fibers has therefore been widely adopted as a primary strategy to improve fiber–matrix bonding. Among various treatments, alkali (NaOH) treatment remains one of the most effective and industrially viable approaches, as it removes surface impurities such as waxes and hemicellulose, promotes fibrillation, and increases surface roughness, thereby enhancing mechanical interlocking with the matrix^7,8^.

While alkali treatment improves interfacial adhesion at the fiber–matrix level, it does not fully address the inherent limitations of polymer matrices, particularly their relatively low stiffness and crack resistance^9^. In this context, the incorporation of nanoscale fillers, especially carbon-based nanomaterials, has emerged as an effective secondary reinforcement strategy. Multi-walled carbon nanotubes (MWCNTs) are of particular interest due to their exceptionally high aspect ratio, stiffness, and ability to bridge microcracks, thereby delaying crack initiation and propagation^10–12^. When adequately dispersed, MWCNTs can significantly enhance the load-bearing capacity and stiffness of polymer matrices even at low weight fractions^13^. However, the mechanical benefits of CNT incorporation are strongly dependent on dispersion quality and interfacial interactions. At low concentrations, well-dispersed CNTs contribute to improved stress transfer and crack-bridging mechanisms, whereas excessive CNT loading often leads to agglomeration, stress concentration, and premature failure^14^. This results in a non-linear and often optimal reinforcement window, beyond which further CNT addition becomes detrimental. Despite numerous studies reporting strength enhancement in CNT-modified natural fiber composites, many investigations rely primarily on trend-based interpretations using average values, with limited emphasis on statistical significance, scatter, or reliability of mechanical performance.

Furthermore, microstructural evidence such as scanning electron microscopy (SEM) is frequently used only for qualitative support, without systematic correlation to quantified mechanical behavior^15^. As a result, the structure–property relationships governing the combined effects of fiber surface modification and nanoscale matrix reinforcement remain insufficiently understood, particularly for bio-based epoxy systems reinforced with sisal fibers^16–18^. In this study, a systematic two-stage reinforcement strategy is adopted to enhance the tensile performance of sisal fiber-reinforced bio-epoxy composites. In the first stage, the influence of alkali treatment on fiber–matrix interfacial efficiency is evaluated by varying NaOH concentration and assessing its effect on tensile strength and stiffness. In the second stage, MWCNTs are incorporated into the bio-epoxy matrix at low weight fractions to identify an optimal CNT content that maximizes mechanical performance without compromising ductility. Unlike conventional studies, the mechanical response is analyzed using statistically robust methods, including confidence intervals and comparative significance analysis, to ensure reliable interpretation of property enhancements.

In addition to conventional tensile metrics, the study emphasizes microstructural–mechanical correlation through detailed SEM analysis of fracture surfaces to elucidate damage mechanisms such as fiber pull-out, matrix cracking, and CNT agglomeration. By integrating mechanical testing with statistical evaluation and microstructural interpretation, this work aims to establish a clear structure–property framework for designing high-performance, bio-based natural fiber composites reinforced through combined chemical and nanoscale modification.

## Background study

Natural fiber–reinforced polymer composites have been widely investigated to address sustainability and weight reduction demands, though their mechanical performance is often limited by weak fiber-matrix interfaces and matrix-dominated failure mechanisms. Several studies have therefore focused on reinforcement optimization through fiber treatment, hybridization, and nanoscale modification.

Based on Sosiati et al.^19^, sisal-based polymer composites reinforced with carbon fibers were studied to evaluate tensile and flexural performance. The authors reported that increasing reinforcement content enhanced stiffness and strength; however, excessive reinforcement caused stress concentration and reduced elongation, highlighting the need for optimized reinforcement levels rather than monotonic addition. The effect of irradiation-assisted modification was explored in the study conducted by Abdel-Hakim et al.^20^, where sisal fiber–reinforced polyester composites were subjected to 5 kGy gamma irradiation along with MWCNT incorporation. The study reported measurable improvements in mechanical and acoustical properties due to enhanced matrix crosslinking and improved fiber–matrix interaction, while excessive CNT loading resulted in performance degradation due to agglomeration.

Processing route effects were investigated by Bosquetti et al.^21^, where sisal fiber composites fabricated using vacuum infusion with castor-oil-based polyurethane resin showed improved tensile performance and reduced void content compared to conventional fabrication routes. The work emphasized the role of resin flow and impregnation quality in controlling mechanical response. A systematic CNT optimization in sisal–epoxy systems was reported by Rao et al.^22^, where composites containing 15 wt% sisal fiber and 0–2.0 wt% CNT were evaluated. The authors identified 1.0 wt% CNT as the optimal content for maximizing thermomechanical and dynamic mechanical properties, with higher CNT fractions leading to reduced efficiency due to agglomeration. In the study by Sahoo et al.^23^, sisal fiber–reinforced polymer composites were examined across varying fiber contents, showing that tensile strength increased up to an optimal fiber loading, beyond which fiber crowding and incomplete wetting reduced mechanical performance.

The role of nanoscale reinforcement was further examined by Muthukumar et al.^24^, which investigated nano-sisal fiber–reinforced polymer composites using SEM, FE-SEM, HR-TEM, and FTIR analysis. The study highlighted the influence of nanoscale features on mechanical and thermal performance, although property optimization remained dependent on dispersion quality. Hybrid nanofiller strategies were explored by Sathish et al.^25^, where basalt/glass fiber composites containing 1–2 wt% hybrid MWCNT–SiO₂ fillers were optimized using response surface methodology. The study demonstrated statistically significant improvements in tensile strength and elongation at optimized filler contents, underscoring the importance of multivariable optimization. Similarly, Boobalan et al.^26^ reported improved mechanical performance in fiber-reinforced composites modified with hybrid nanofillers, identifying an optimal nanoparticle concentration range beyond which agglomeration adversely affected tensile behavior.

Short sisal fiber biocomposites incorporating low CNT fractions were studied by Pantano et al.^27^, where CNT addition improved tensile strength and fatigue resistance by enhancing matrix toughness and fiber–matrix bridging, as confirmed through SEM fracture analysis. Statistical rigor was emphasized by DOI: Sathish et al.^28^, where polypropylene/sisal/Kevlar composites modified with SiO₂ nanoparticles demonstrated statistically significant improvements in tensile and flexural properties (*p* < 0.05), supported by power analysis at 80% confidence. A comprehensive optimization study using Box–Behnken design was presented by Nor et al.^29^, where kenaf/MWCNT composites were optimized by varying fiber loading, NaOH concentration, CNT content, and sonication time. Strong interaction effects were reported, confirming the coupled influence of surface treatment and nano-reinforcement.

CNT-modified natural fiber composites were further studied by Satish et al.^30^, where sisal–jute hybrid composites containing CNTs showed enhanced tensile strength and thermal stability, though increased CNT content led to reduced ductility. In a study by Sathishkumar et al.^31^, sisal fiber–reinforced polyester composites with fiber contents of 5–20 wt% were evaluated. The study reported that 15 wt% fiber provided the best balance of tensile, flexural, and damping properties, supported by SEM observations of reduced fiber pull-out. Layered hybrid architectures were explored by Arumugam et al.^32^, where glass/sisal/epoxy sandwich composites demonstrated improved tensile and flexural response due to controlled layer sequencing and stress redistribution.

MWCNT-reinforced hybrid natural fiber composites were studied by Prabhudass et al.^33^, where bamboo/kenaf composites exhibited an ~ 80.6% improvement in impact strength, attributed to CNT-induced crack deflection and energy absorption. In the research conducted by Loganathan et al.^34^, hybrid phenolic composites reinforced with 0.5 wt% MWCNT and varying natural fiber ratios demonstrated improved tensile and flexural properties, with optimal performance depending on fiber hybridization. Alkali-treated sisal fiber composites reinforced with halloysite nanotubes were examined by Krishnaiah et al.^35^, where high-intensity sonication improved filler dispersion and interfacial bonding, resulting in enhanced mechanical properties. The influence of CNT addition on multiple mechanical properties was reported by Swikker et al.^36^, where small CNT additions improved tensile, compressive, flexural, and impact strength in fiber-reinforced composites.

Hybrid sisal/glass epoxy laminates incorporating MXene and CNTs were investigated by Bodduru et al.^37^, demonstrating enhanced tensile and bending performance due to synergistic nano-reinforcement effects. Eco-friendly chemical surface modification was reported by Behera et al.^38^, where sodium citrate and stearic acid treatments of sisal fibers improved mechanical, thermal, and tribological performance of epoxy composites. MWCNT-enriched hybrid laminates were examined by Arunachalam et al.^39^, reporting improved mechanical and thermal behavior alongside reduced moisture absorption. Compression-molded hybrid composites containing 0.3–0.9 wt% MWCNT were optimized by Rangaswamy et al.^40^, where an optimal CNT content maximized tensile and tribological performance. Finally, Bodduru et al.^41^ demonstrated that sisal/glass epoxy laminates modified with MXene, graphene nanoplatelets, and MWCNTs exhibited improved tensile strength, reduced swelling, and enhanced flammability resistance, though dispersion control remained critical.

Recent studies consistently report that chemical treatment enhances interfacial bonding in natural fiber composites, while nanoscale fillers improve stiffness and strength when dispersion is well controlled. However, excessive nanofiller loading often introduces agglomeration and variability, limiting mechanical gains. The reviewed literature also highlights the strong influence of fiber architecture and processing routes on tensile performance. The discussions in Table 1 align with these trends, where alkali treatment improved load transfer and an optimum CNT content (0.25 wt%) provided the best strength-stiffness balance without severe agglomeration effects.

**Table 1:** Comparative summary of reported literatures with the present study.

Ref	System (matrix + reinforcement + nano)	Treatment/key processing (as reported)	Fiber/filler amount	Key mechanical metrics extracted (most comparable)	Relevance
This study	Bio-based epoxy + sisal woven mat; NaOH (0, 4, 5 wt%); MWCNT (0–0.35 wt%)	Hand lay-up + light compression; ambient cure > 24 h; no post-cure	Vf ≈ 30.2–31.2%; Wf ≈ 37.5–38.5%; void ≈ 2.5–3.8%	UTS: 71.24 → 103.32 MPa (NaOH), peak 129.36 MPa at 0.25% CNT; E: 4.8 → 6.9 GPa (NaOH), peak 8.1 GPa (0.25% CNT); εf: 2.95–3.05% (no CNT) → ~1.15–1.5% (with CNT); Toughness: 1.95 → 2.80 MJ·m⁻³ (NaOH) then ~ 1.10–1.40 MJ·m⁻³ (with CNT)	Alkali treatment improves fiber–matrix bonding and tensile load transfer.
^42^	Epoxy + PALF (uni/bidirectional); GO functionalization (study focus)	(Paper focus: impact/ballistic)	30 vol% PALF explicitly mentioned	Izod impact absorbed energy = 287.70 J/m (reported for untreated composite containing 30 vol% unidirectional PALF)	Nanofiller benefits occur at low loading but decline with agglomeration.
^43^	Epoxy + ramie fabric, with graphene oxide in matrix (study focus: ballistic/impact)	GO incorporated; ballistic velocity measured (chronographs); GO dispersion step includes sonic shaker 2 h at 45 °C (reported)	Not cleanly extractable as Vf from text snippet	Impact/ballistic absorbed energy ~ 111.51–112.77 J (values explicitly stated)	Hybrid fiber architecture strongly influences tensile performance.
^44^	Epoxy + 20 vol% mallow fibers (study focus: curing kinetics)	DSC-based curing kinetics (multiple heating rates; kinetics vs. temperature)	20 vol% fiber	Reports curing kinetic parameters (activation energy/pre-exponential/time-to-50%-cure vs. temperature), not tensile	Chemical treatment enhances stiffness and interfacial adhesion.
^45^	DGEBA/TETA epoxy + fique fabric (study focus: water-immersion aging; DMA/morphology)	Fabric dried 70 °C ≥ 24 h; compression molding; laminate ~ 3.2 mm (reported)	40 vol% fabric composite (reported)	From fiber property table: fique fiber tensile strength = 132.4–237 MPa, elongation at break = 6–9.8%	CNTs improve strength and modulus when dispersion is well controlled.
^46^	DGEBA/TETA epoxy + 30 vol% arapaima scales (focus: flexural)	3-point bending	30 vol% scales	Flexural strength: plain epoxy 71.6 ± 25.5 MPa, composite 43.6 ± 10.9 MPa; Flexural modulus: plain epoxy 2.82 ± 0.49 GPa, composite 4.50 ± 0.70 GPa	Nanographene improves properties when uniformly dispersed.
^47^	Modified kenaf FRP + nanographene, with ANN-CCD modelling	ANN + CCD optimization; model fit reported	Nanographene contents of 0, 1, and 2 wt%	Optimum tensile strength predicted = 46 MPa, experimentally 45.4 MPa (~ 98.45% prediction accuracy); R² = 0.9805	Hybrid nanofillers require strict dispersion control for gains.
^48^	Epoxy + hybrid jute/kenaf/glass; MWCNT (0–3 wt%) + nanographene (0 or 3 wt%); KMnO₄ treated hybrid fiber	Mixing 150 rpm; compression molding 140 °C, 150 MPa, 1–2 min; panel 300 × 300 × 3 mm	Fiber 45 wt%, epoxy 55 wt% (formulations table)	Tensile strength 101–128 MPa (max 128 MPa); Flexural strength up to 152 MPa; fracture toughness ~ 2.08–2.57 (MPa·m^0.5); hardness 74–91 HV	High filler loadings can improve strength but may increase variability.
^49^	Jute/kenaf/glass fiber epoxy with nano-hybridization (focus: flexural/impact)	Automotive application oriented	Used 60 wt% fiber and 5 wt% nanofiller ~ 35 wt% epoxy matrix	Reports: Flexural strength 105 → 136 MPa; Flexural modulus up to 6.844 GPa; Impact strength 123 → 169 J/m	Stacking sequence and processing govern hybrid composite performance.
^50^	Chemically treated jute/kenaf + glass fiber composite with SiO nanoparticles; RSM optimization	RSM/optimization based study	Investigated 3–5 wt% SiO₂ nanofiller	Reports: maximum flexural strength = 147 MPa; mean flexural strength ~ 140 MPa	Alkali treatment improves fiber–matrix bonding and tensile load transfer.

## Materials and methods

### Materials procurement

**Fig. 1:**
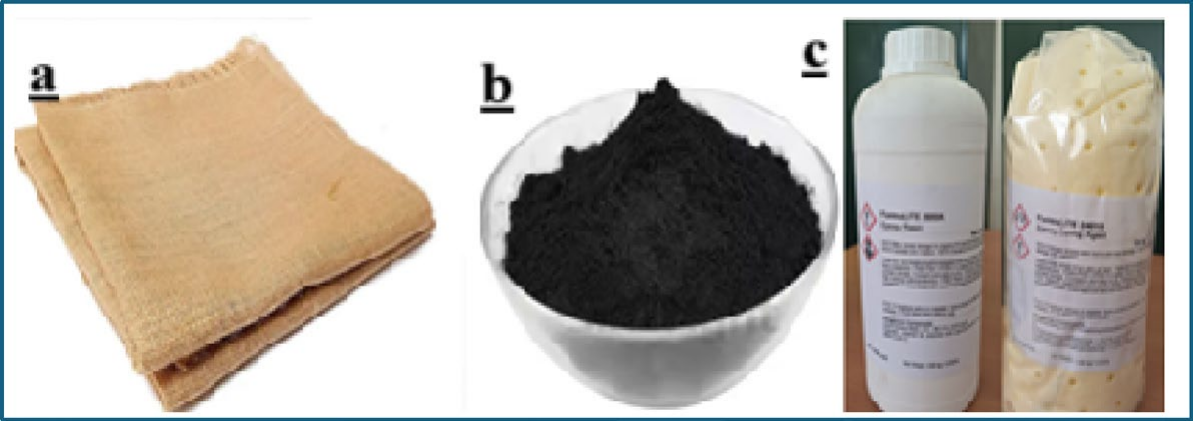
Procured raw materials (**a**) Sisal fiber mat (**b**) MWCNT powder (**c**) Biobased epoxy.

Bidirectional woven sisal fiber mats were procured from a local commercial supplier and used as the primary natural fiber reinforcement in the present study. The mats were supplied in untreated form and were cut into required dimensions prior to surface modification. A bio-based epoxy resin system was employed as the matrix material to align with the sustainability objectives of the work. The epoxy resin and its corresponding curing agent (hardener) were supplied by the manufacturer in liquid form and used as received. Multi-walled carbon nanotubes (MWCNTs) were used as nanoscale secondary reinforcement for matrix modification. The MWCNTs were procured in powder form and characterized by high aspect ratio and high purity, as detailed in the material properties table. All chemicals used for fiber treatment were of analytical grade and obtained from standard laboratory suppliers. Figure 1 illustrates the materials procured for composite fabrication, including the sisal fiber mat, epoxy resin system, and MWCNTs.

The bio-based epoxy resin used in this study served as the continuous matrix phase, providing load transfer and structural integrity to the composite. The resin was selected based on its compatibility with natural fibers, adequate mechanical strength, and curing behavior suitable for composite fabrication under laboratory conditions. The epoxy resin was mixed with its corresponding hardener in the manufacturer-recommended ratio to ensure complete crosslinking. The fundamental physical and mechanical properties of the epoxy resin system used in this research are summarized in Table 2.

**Table 2:** Properties of bio based epoxy CNSL FormuLITE.

Parameters	Formulite 2500 A
Key advantages	Good Tg, potlife, bio-content and mechanical properties
Calculated bio-content	36.6%
Mix ratio by weight	100:30
Mix ratio by volume	100:36
Mix viscosity 25 °C (cPs)	700
Pot life at 25 °C (min)	105
Pot life at 40 °C (min)	57
Suggested cure cycles	4–8 h at RT + 2–4 h at 50–70 °C* + 2–3 h at 80–100 °C
Tg (°C)	92
Tensile strength (MPa)	62
Tensile modulus (MPa)	2615
Elongation at Fmax (%) Elongation at break (%)	4.8/6.4
Flexural strength (MPa)	92
Flexural modulus (MPa)	2262
Recommended processes	Infusion, RTM, VARTM, lamination, wet lay-up

MWCNTs were incorporated into the epoxy matrix to enhance matrix stiffness, crack resistance, and load transfer efficiency at the fiber matrix interface. The selected MWCNTs possessed high aspect ratio, nanoscale diameter, and high carbon purity, making them effective for reinforcing polymer matrices at low weight fractions. The key properties of the MWCNTs used in this study are presented in Table 3. These characteristics play a critical role in governing dispersion behavior and reinforcing efficiency within the epoxy matrix.

**Table 3:** Properties of MWCNT.

Property	Description/typical value
Structure	Multiple concentric graphene cylinders
Outer diameter	10–100 nm
Inner diameter	1–5 nm
Length	1–20 μm
Aspect ratio	up to 1000:1
Density	~ 1.3 g/cm³
Tensile strength	40–50 GPa
Young’s modulus	~ 1 TPa (comparable to diamond)
Electrical conductivity	~ 10⁴ S/m (metallic/semiconducting behavior)
Thermal conductivity	~ 3000 W/m·K (higher than copper)
Specific surface area	~ 350 m²/g
Chemical stability	High (inert, resistant to many chemicals)
Thermal stability	Stable up to ~ 600 °C in air, > 2000 °C in inert atmosphere

### Alkali treatment of sisal fiber mats

To improve fiber matrix interfacial adhesion, sisal fiber mats were subjected to alkali treatment using sodium hydroxide (NaOH) solutions. Alkali treatment was carried out at two different concentrations, namely 4 wt% and 5 wt% NaOH, to study the influence of treatment severity on composite performance. The required concentration of NaOH solution was prepared by dissolving sodium hydroxide pellets in distilled water under continuous stirring. The sisal fiber mats were fully immersed in the prepared NaOH solutions and maintained under static conditions to ensure uniform chemical interaction between the fibers and the alkali medium. The treatment duration was kept constant for all samples to isolate the effect of NaOH concentration.

**Fig. 2:**
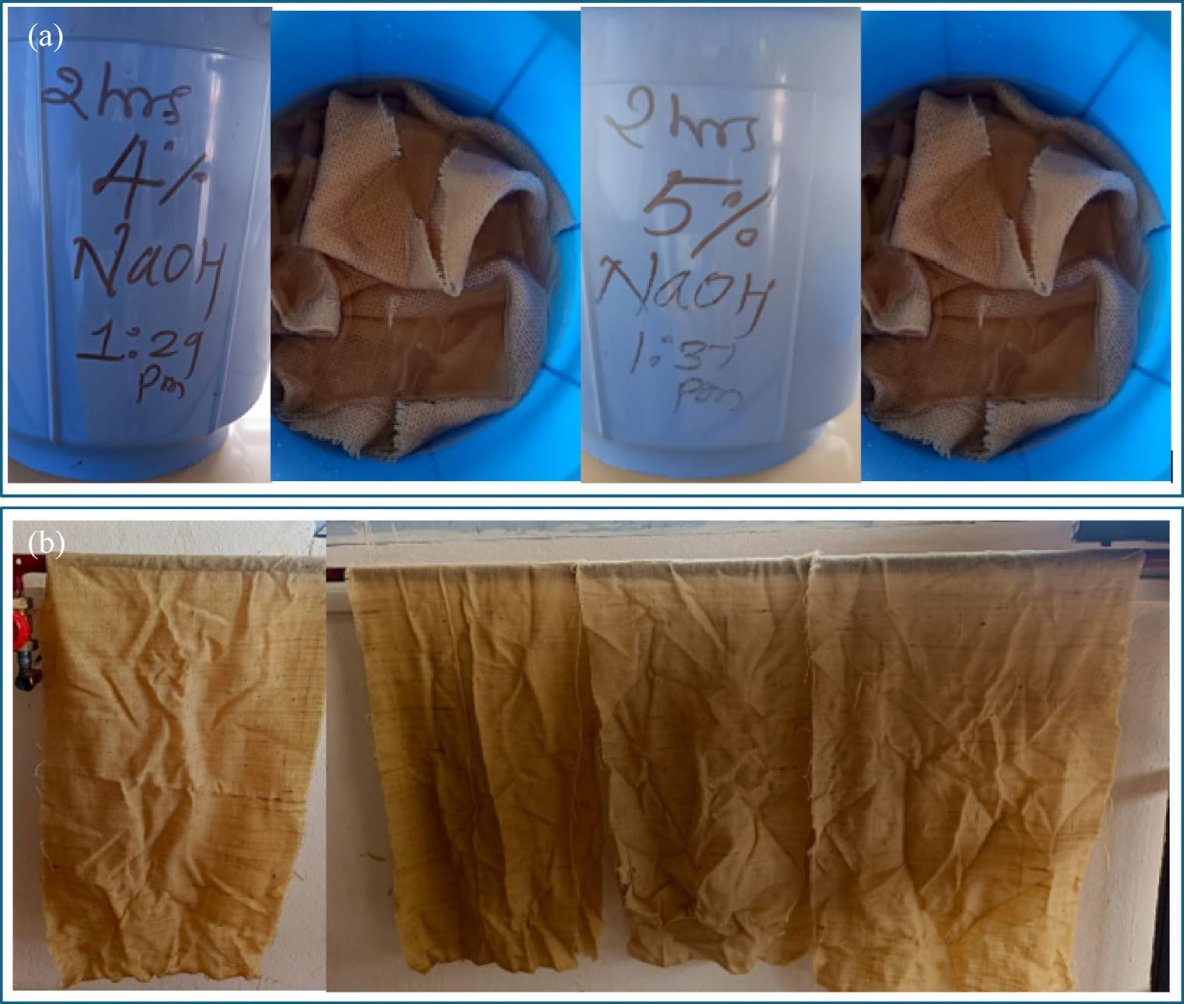
Alkali treatment (**a**) Sisal fiber mats immersion (**b**) Drying process.

During alkali treatment, non-cellulosic components such as hemicellulose, surface waxes, and lignin were partially removed from the fiber surface. This process promotes fibrillation of the fiber bundles, increases surface roughness, and exposes additional hydroxyl groups, thereby enhancing mechanical interlocking and interfacial bonding with the epoxy matrix. Figure 2(a) shows the sisal fiber mats immersed in 4 wt% and 5 wt% NaOH solutions during the alkali treatment process. After completion of alkali treatment, the sisal fiber mats were thoroughly washed with distilled water to remove residual NaOH and reaction by-products from the fiber surface. Washing continued until the rinse water reached near-neutral pH, ensuring the complete removal of alkali traces that could otherwise degrade the fiber structure or interfere with epoxy curing. All fibers and matrix materials were procured from consistent suppliers/batches to reduce material-to-material variability. Although alkali treatment involves chemical processing, rinsing to neutral pH and controlled solution handling were adopted to minimize environmental impact.

The washed fiber mats were then air-dried under ambient laboratory conditions, followed by oven drying at a controlled temperature to remove absorbed moisture (Fig. 2(b)). Proper drying was essential to minimize moisture-induced defects such as void formation and poor interfacial bonding during composite fabrication. The dried mats were stored in sealed containers to prevent moisture uptake prior to composite processing. After drying the mats were kept under compression load to remove wrinkles. Sisal woven mats were treated in 4 wt% and 5 wt% NaOH solutions by immersion for 4 h at room temperature (~ 25 °C) using a bath-to-fiber ratio of approximately 20:1. Gentle periodic agitation ensured uniform treatment. After immersion, the mats were rinsed with distilled water until neutral pH was reached and then oven-dried at 60 °C for 24 h. The dried mats were subsequently flattened under light compression (~ 0.2 MPa) for 12 h prior to composite fabrication.

### Composite fabrication of sisal fiber–reinforced epoxy and MWCNT-modified composites

**Fig. 3:**
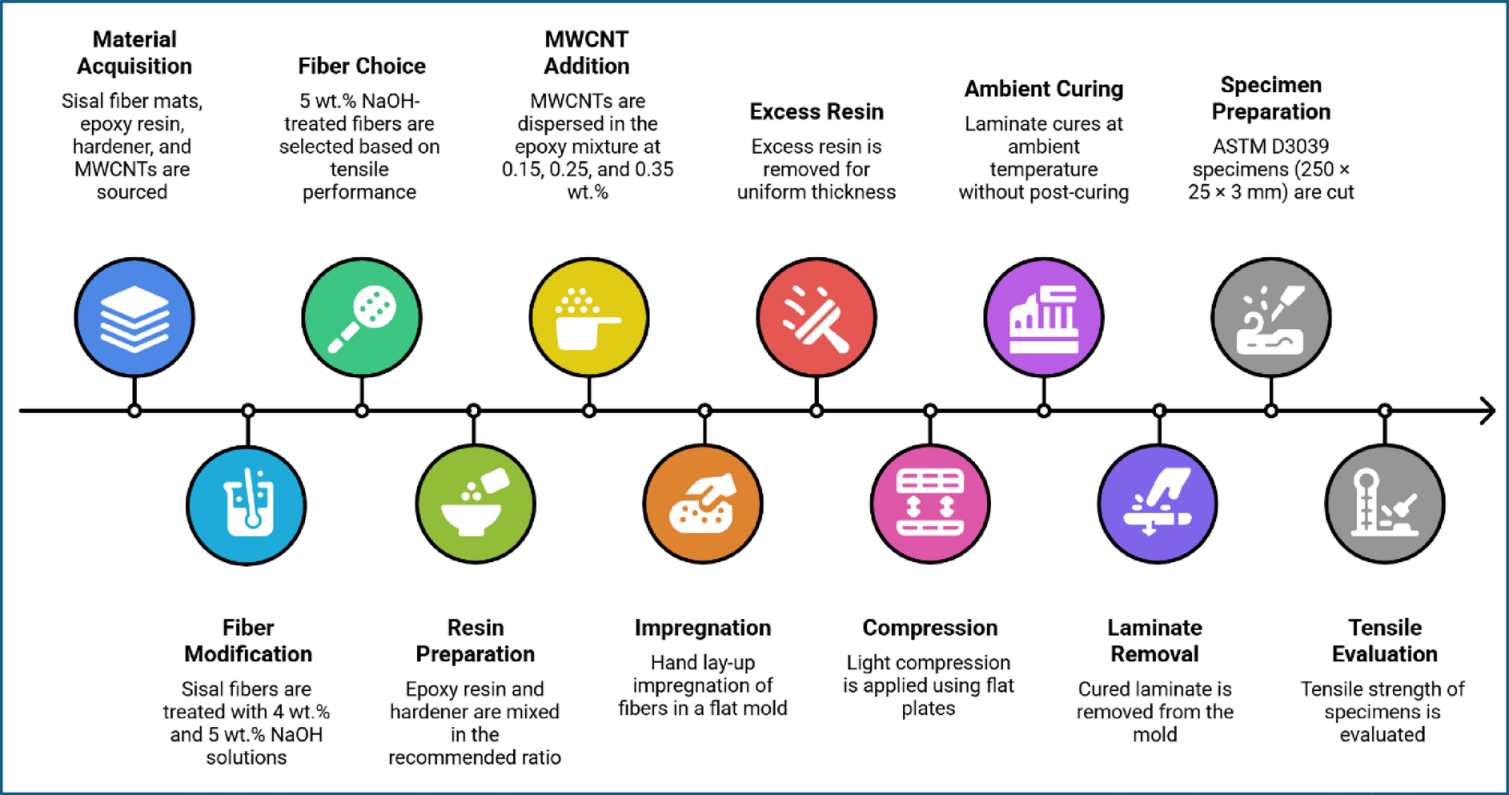
Sisal fiber composite fabrication and testing process.

**Fig. 4:**
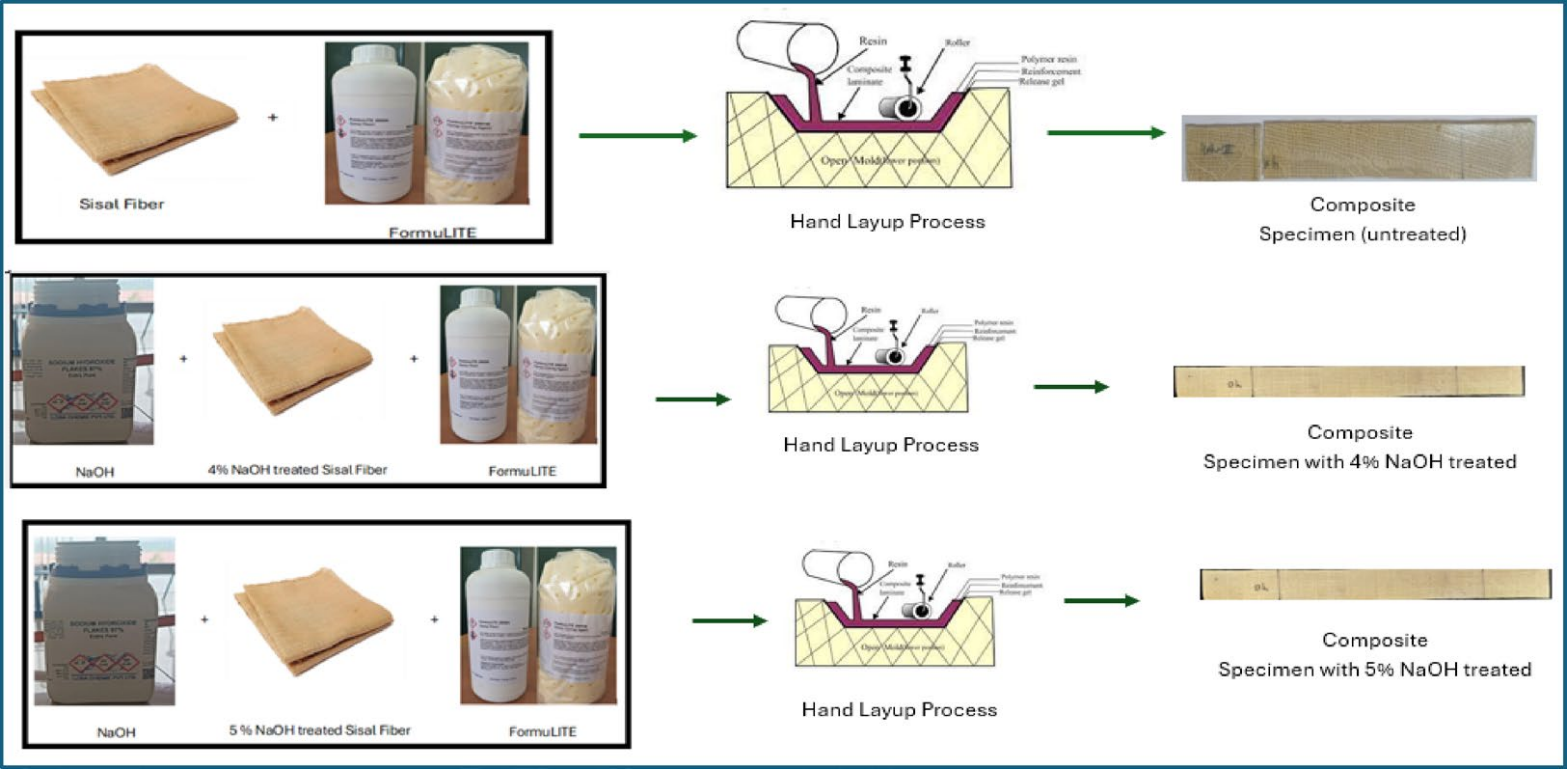
Alkali treated specimen preparation for tensile test as per ASTM D3039.

Figure 3 demonstrates the workflow of the composite fabrication process. Initially composite laminates were fabricated using a controlled hand lay-up technique followed by consolidation and ambient curing. In the first stage of the study, sisal fiber mats in three different conditions untreated, 4 wt% NaOH-treated, and 5 wt% NaOH-treated were employed to prepare baseline epoxy composites in order to evaluate the influence of fiber surface modification on composite performance. The epoxy resin and hardener were mixed thoroughly in the manufacturer-recommended stoichiometric ratio prior to impregnation. The prepared resin system was uniformly applied onto the sisal fiber mats placed inside a flat mold, ensuring complete wetting of the fibers and homogeneous resin distribution throughout the laminate. Excess resin was carefully removed to maintain consistent laminate thickness and fiber volume fraction. All laminates were cured under ambient laboratory conditions (25 ± 2 °C) for 24 + h followed by conditioning at room temperature. Following impregnation, the laminates were subjected to light compression using a flat plate arrangement to reduce void content and improve fiber–matrix contact. The composites were cured under ambient laboratory conditions, and no post-curing treatment was applied. Figure 4 shows the fabrication stages of epoxy composites reinforced with untreated, 4 wt% NaOH-treated, and 5 wt% NaOH-treated sisal fiber mats. Tensile test specimens were cut from the laminates in accordance with ASTM D3039. The specimens were prepared with dimensions of 250 × 25 × 3 mm (length × width × thickness).

Based on the tensile performance obtained from the alkali treatment study, 5 wt% NaOH-treated sisal fiber mats were selected for further modification using CNT-reinforced epoxy matrices. To enhance the matrix-dominated mechanical response, multi-walled carbon nanotubes (MWCNTs) were incorporated into the epoxy resin at low weight fractions of 0.15 wt%, 0.25 wt%, and 0.35 wt%, calculated with respect to the epoxy resin prior to hardener addition. MWCNTs were first mixed into the epoxy resin using mechanical stirring at 800 rpm for 30 min to achieve preliminary wetting at room temperature, followed by controlled ultrasonication in pulsed mode with intermittent cooling to break down CNT agglomerates while limiting temperature rise and viscosity variation. The mixture was then subjected to probe ultrasonication (20 kHz) in pulsed mode (5 s on/5 s off) for 20 min at ~ 40% amplitude. Intermittent cooling using an ice bath was applied to maintain the resin temperature below 40 °C and prevent premature curing. After achieving a visually uniform dispersion, the hardener was added in the recommended ratio and mixed gently to minimize air entrapment. The CNT-modified epoxy system was used immediately for laminate fabrication to prevent sedimentation effects. Later, Dispersion quality was assessed indirectly through fracture surface SEM observations of the cured composites.

**Fig. 5:**
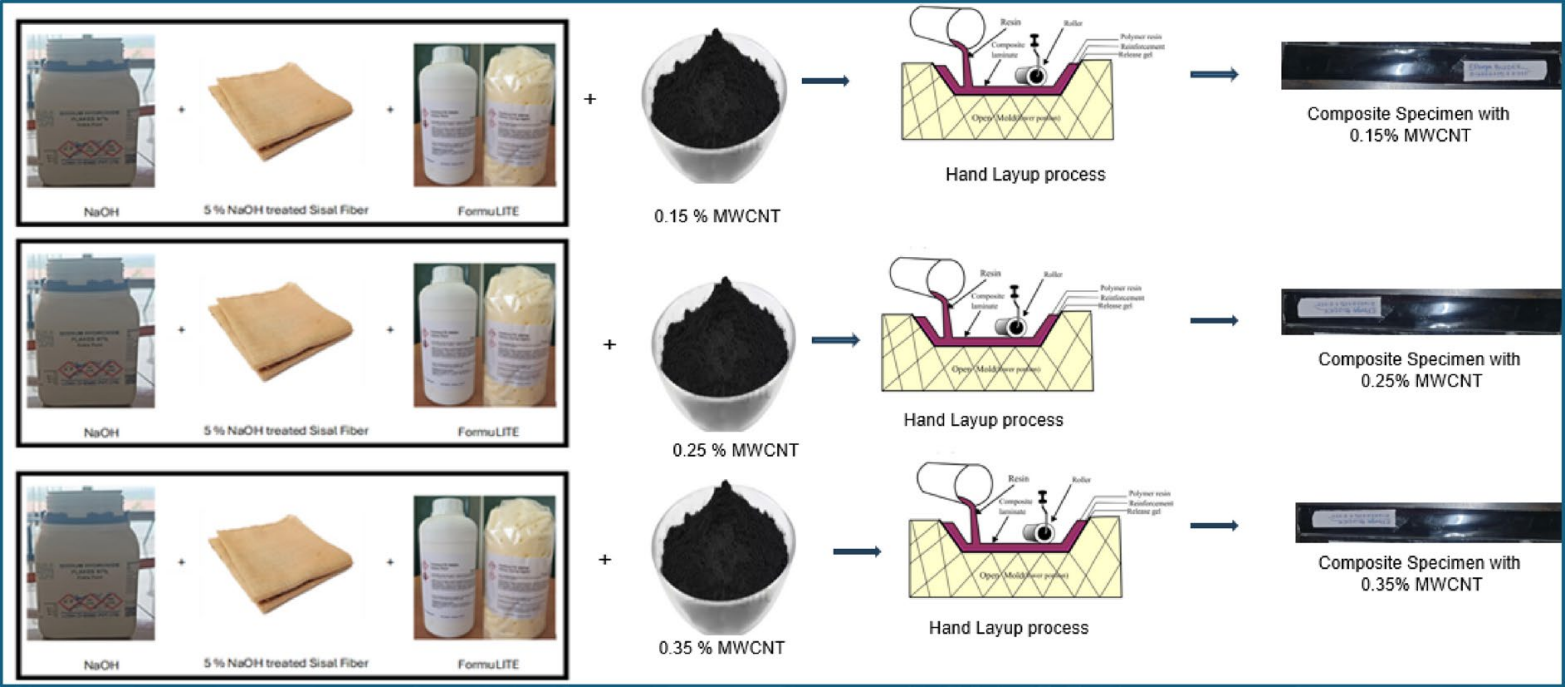
Tensile test specimen fabrication process with MWCNT filler.

The 5 wt% NaOH-treated sisal fiber mats were impregnated with the CNT-modified epoxy resin using the same hand lay-up procedure adopted for the baseline composites. Excess resin removal, consolidation, and ambient curing conditions were kept identical to those used for the untreated and alkali-treated composites to ensure consistency across all formulations. Figure 5 presents the fabrication sequence of 5 wt% NaOH-treated sisal fiber composites incorporating different MWCNT weight fractions.

All laminates were fabricated using identical processing conditions to ensure reproducibility. The reinforcement consisted of bidirectional woven sisal mats with an aerial weight of approximately 300 g/m² (manufacturer specification), arranged in a fixed four-ply symmetric lay-up for all composite systems. Excess resin was removed during hand lay-up to achieve uniform impregnation, and laminates were consolidated under flat-plate compression during ambient curing. Laminate thickness and density were measured at the specimen level, and fiber and void fractions were determined using density-based rule-of-mixtures calculations commonly applied to natural fiber composites. The resulting physical parameters for S1–S6 are summarized in Table 4.

**Table 4:** Laminate manufacturing and physical parameters of S1–S6 composites.

Sample ID	Formulation	Thickness (mm)	Density (g/cm³)	Fiber Vol.% (Vf)	Fiber Wt.% (Wf)	Void Content (%)
S1	Untreated sisal	3.12 ± 0.08	1.18 ± 0.02	31.2	38.5	3.8 ± 0.4
S2	4% NaOH treated	3.08 ± 0.05	1.19 ± 0.01	30.9	38.2	3.2 ± 0.3
S3	5% NaOH treated	3.05 ± 0.04	1.20 ± 0.02	30.7	38	2.9 ± 0.2
S4	5% NaOH + 0.15% MWCNT	3.03 ± 0.03	1.21 ± 0.01	30.5	37.8	2.7 ± 0.2
S5	5% NaOH + 0.25% MWCNT	3.01 ± 0.02	1.22 ± 0.01	30.3	37.6	2.5 ± 0.1
S6	5% NaOH + 0.35% MWCNT	3.02 ± 0.04	1.22 ± 0.02	30.2	37.5	3.1 ± 0.3

### Mathematical modelling and data analysis-tensile property calculations (ASTM D3039)

Tensile properties of the composites were evaluated in accordance with ASTM D3039. The experimentally measured load–displacement data were converted into engineering stress and strain using the specimen’s initial cross-sectional area and gauge length. The ultimate tensile strength (UTS) was defined as the maximum stress attained before fracture, while the elastic modulus was obtained from the slope of the initial linear region of the stress–strain curve. The strain at break was determined at the point of specimen failure. In addition, the toughness of the composites was calculated as the area under the stress–strain curve up to fracture to assess energy absorption behaviour. The averaged tensile parameters were subsequently used for comparative analysis and mathematical modelling.

Engineering stress and strain were computed as:

$$\sigma=\frac{F}{{A}_{0}},\epsilon=\frac{{\Delta}L}{{L}_{0}}$$

where $F$is the applied load, ${A}_{0}$ is the initial cross-sectional area of the specimen, ${\Delta}L$ is the extension, and ${L}_{0}$ is gauge length.

- Ultimate Tensile Strength (UTS):
  $${\sigma}_{\mathrm{UTS}}=\mathrm{m}\mathrm{a}\mathrm{x}\left(\sigma\right)$$
- Elastic Modulus (E):
  determined from the slope of the linear elastic region of the engineering stress-strain curve:
  $$E=\frac{{\Delta}\sigma}{{\Delta}\epsilon}$$
- Elongation at Break:
  $${\epsilon}_{f}=\epsilon\mathrm{\:\:at\:fracture}$$
- Toughness (strain energy density): computed as the area under the engineering stress-strain curve up to fracture.
  $$U={\int}_{0}^{{\epsilon}_{f}}\sigma\left(\epsilon\right){\hspace{0.17em}}d\epsilon$$
  (implemented numerically from discrete data using the trapezoidal integration).

### Replicates, uncertainty, and comparative metrics

For each formulation (untreated, 4% and 5% NaOH treated; and 0.15/0.25 and 0.35 wt% MWCNT), the results were reported in terms of mean and dispersion:

$$\bar{x}=\frac{1}{n}\sum_{i=1}^{n}{x}_{i}$$

$$s=\sqrt{\frac{\sum_{i=1}^{n}({x}_{i} - \bar{x}{)}^{2}}{n-1}}$$

- Coefficient of Variation (CV%):
  $$CV\left(\mathrm{\%}\right)=\frac{s}{\stackrel{\prime }{x}}\times100$$
- 95% Confidence Interval (CI) for the Mean:
  $$C{I}_{95\mathrm{\%}}=\stackrel{\prime }{x}\pm{t}_{0.975,{\hspace{0.17em}}n-1}\left(\frac{s}{\sqrt{n}}\right)$$
- Percent Improvement (relative to the reference group, e.g., untreated or neat epoxy):
  $$\mathrm{\%}{\Delta}=\frac{{x}_{\mathrm{new}}-{x}_{\mathrm{ref}}}{{x}_{\mathrm{ref}}}\times100$$

### Composition–property modelling (MWCNT optimization)

To quantify the effect of (CNT) weight fraction $C$ on the tensile response and identify an optimum, a quadratic regression model was adopted:

$$y=a+bC+c{C}^{2}$$

where $y$ represents ${\sigma}_{\mathrm{UTS}}$, $E$, or $U$. The optimum (CNT) content (for $c<0$) was determined from:

$${C}^{*}=-\frac{b}{2c}$$

Model Performance was reported using:

$${R}^{2}=1-\frac{\sum({y}_{i}-{\widehat{y}}_{i}{)}^{2}}{\sum({y}_{i}-\stackrel{\prime }{y}{)}^{2}}$$

$$RMSE=\sqrt{\frac{1}{n}\sum({y}_{i}-{\widehat{y}}_{i}{)}^{2}}$$

### Reliability analysis (optional but strong): Weibull modelling of tensile strength

The scatter was analysed using a two-parameter Weibull distribution:

$${P}_{f}\left(\sigma\right)=1-\mathrm{exp}\left[-{\left(\frac{\sigma}{{\sigma}_{o}}\right)}^{m}\right]$$

where ${P}_{f}$ denotes the probability, $m$ is the Weibull modulus (scatter/reliability), and ${\sigma}_{0}$is the characteristic strength linearization was performed as:

$$\mathrm{ln}\left[\mathrm{ln}\left(\frac{1}{1-{P}_{f}}\right)\right]=\mathrm{mln}\sigma-\mathrm{mln}{\sigma}_{0}$$

with ${P}_{f}$ estimated from ranked strengths (median rank):

$${P}_{f}=\frac{i-0.3}{n+0.4}$$

$i$ is the rank,$n$is the total number of specimens.

Performance Index (PI)

$$PI={w}_{1}{\widehat{\sigma}}_{\mathrm{UTS}}+{w}_{2}\widehat{E}+{w}_{3}\widehat{U}+{w}_{4}{\widehat{\epsilon}}_{f}$$.

## Results and discussion

### Tensile stress–strain behaviour of sisal fiber composites

**Fig. 6:**
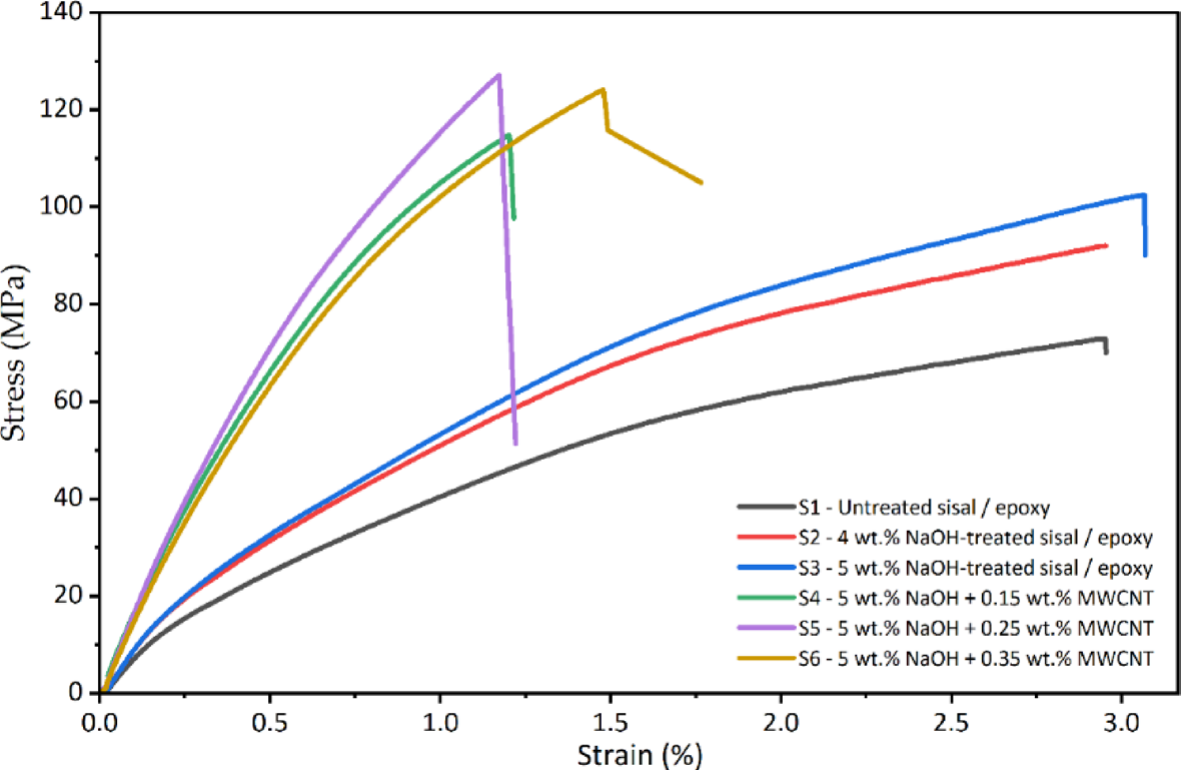
Stress strain responses of all six composites.

Figure 6 presents the representative stress–strain responses of all six composite formulations, including untreated, alkali-treated, and MWCNT-modified sisal fiber–reinforced epoxy composites. The curves correspond to specimens whose ultimate tensile strength lies close to the average value obtained from five tested samples in each category, ensuring representative mechanical behaviour. The untreated composite exhibits a relatively low initial slope and an extended strain-to-failure, indicating limited load transfer efficiency and premature interfacial debonding between the hydrophilic sisal fibers and the epoxy matrix. The gradual stress increase followed by early failure reflects weak fiber–matrix adhesion and dominant fiber pull-out mechanisms. Alkali treatment significantly alters the stress–strain response. The 4 wt% NaOH-treated composite shows a noticeable increase in both the initial slope and peak stress compared to the untreated composite, indicating improved interfacial bonding and enhanced stress transfer. Further improvement is observed for the 5 wt% NaOH-treated composite, which exhibits the steepest elastic slope among the CNT-free samples and a higher ultimate tensile stress, confirming the effectiveness of alkali treatment in removing surface impurities and increasing fiber surface roughness.

The incorporation of MWCNTs into the epoxy matrix further modifies the tensile response of the 5 wt% NaOH-treated composites. At 0.15 wt% MWCNT, the stress–strain curve shows a moderate increase in stiffness and peak stress with reduced strain at failure, indicating matrix stiffening without severe embrittlement. The composite containing 0.25 wt% MWCNT exhibits the highest peak stress and a sustained load-bearing response prior to fracture, suggesting effective CNT dispersion and crack-bridging behaviour. In contrast, the 0.35 wt% MWCNT composite shows a slight reduction in peak stress and altered post-yield behaviour, indicating the onset of CNT agglomeration and localized stress concentrations. The composite specimens were assigned a ID between S1 to S6 and the assigned nomenclature is discussed in Table 4.

### Effect of alkali treatment on ultimate tensile strength

**Fig. 7:**
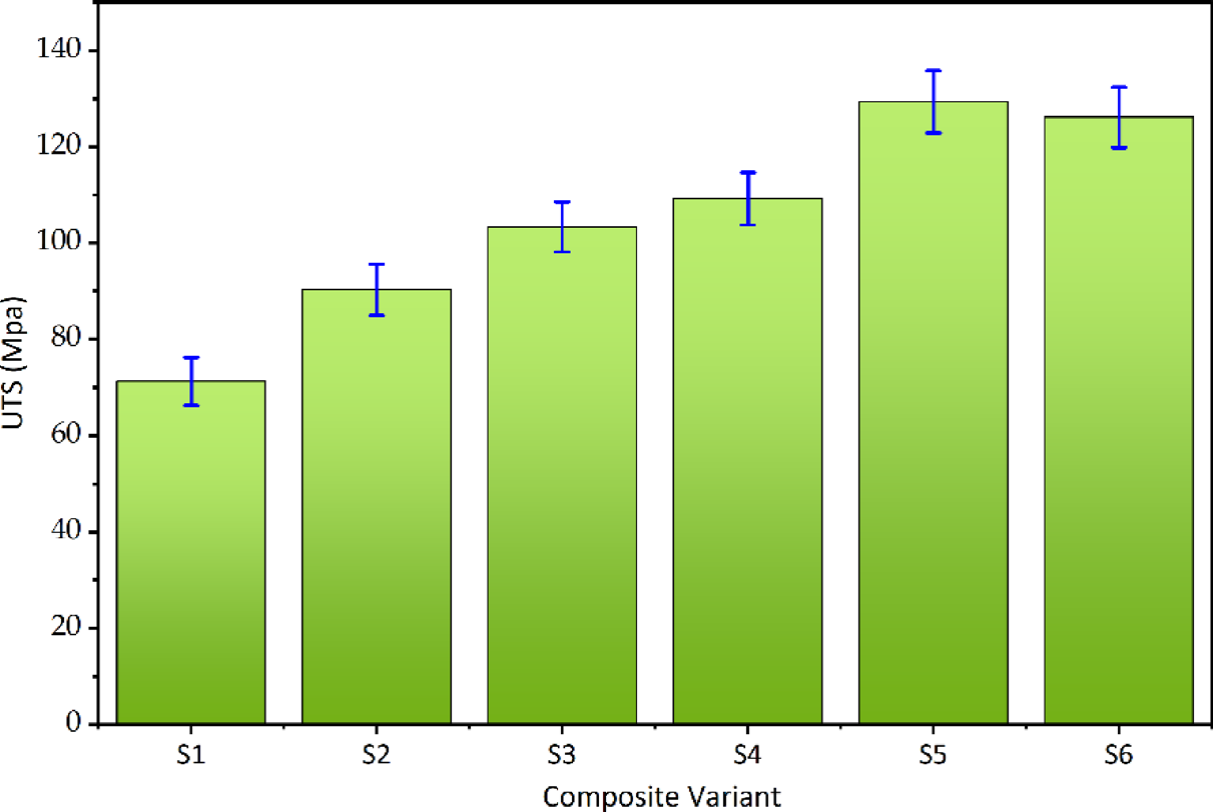
Variation in ultimate tensile strength (UTS) across five repeated tensile tests.

The influence of alkali treatment on the tensile strength of sisal fiber–reinforced epoxy composites is summarized in Fig. 7; Table 5. A clear and systematic improvement in ultimate tensile strength (UTS) is observed with increasing NaOH concentration, confirming the critical role of fiber surface modification in governing load transfer efficiency. The untreated composite exhibits the lowest tensile strength, with a UTS of 71.24 ± 5.00 MPa, which can be attributed to poor interfacial adhesion between the hydrophilic sisal fibers and the epoxy matrix. In the absence of surface treatment, the presence of surface waxes, lignin, and hemicellulose limits mechanical interlocking, leading to premature interfacial debonding and fiber pull-out during tensile loading. The elastic modulus was obtained from the initial linear region under constant strain rate; while sisal composites are viscoelastic, identical testing conditions across samples ensure valid relative comparison.

Upon treatment with 4 wt% NaOH, the UTS increases significantly to 90.26 ± 5.30 MPa, corresponding to an improvement of approximately 26.7% relative to the untreated composite. This enhancement indicates effective removal of surface impurities and partial fibrillation of the fiber bundles, which increases surface roughness and promotes better stress transfer across the fiber–matrix interface. A further increase in NaOH concentration to 5 wt% results in a UTS of 103.32 ± 5.17 MPa, representing an overall improvement of approximately 45.0% compared to the untreated composite and 14.5% compared to the 4 wt% NaOH-treated composite. The relatively narrow error margins across the three conditions suggest good experimental repeatability and indicate that the observed improvements are systematic rather than incidental. The progressive increase in tensile strength with alkali concentration demonstrates that 5 wt% NaOH treatment provides a more effective interfacial modification than 4 wt%, without inducing fiber damage or degradation.

**Table 5:** Elastic modulus and strain at break derived from representative stress–strain curves.

Sample ID	Composite description	Elastic modulus, E (GPa)	Strain at break, εf (%)
S1	Untreated sisal/epoxy	4.8 ± 0.3	2.95 ± 0.12
S2	4 wt% NaOH-treated sisal/epoxy	6.2 ± 0.4	2.90 ± 0.10
S3	5 wt% NaOH-treated sisal/epoxy	6.9 ± 0.3	3.05 ± 0.11
S4	5 wt% NaOH + 0.15 wt% MWCNT	7.4 ± 0.4	1.20 ± 0.08
S5	5 wt% NaOH + 0.25 wt% MWCNT	8.1 ± 0.3	1.15 ± 0.07
S6	5 wt% NaOH + 0.35 wt% MWCNT	7.9 ± 0.5	1.50 ± 0.09

### Effect of MWCNT content on ultimate tensile strength

**Fig. 8:**
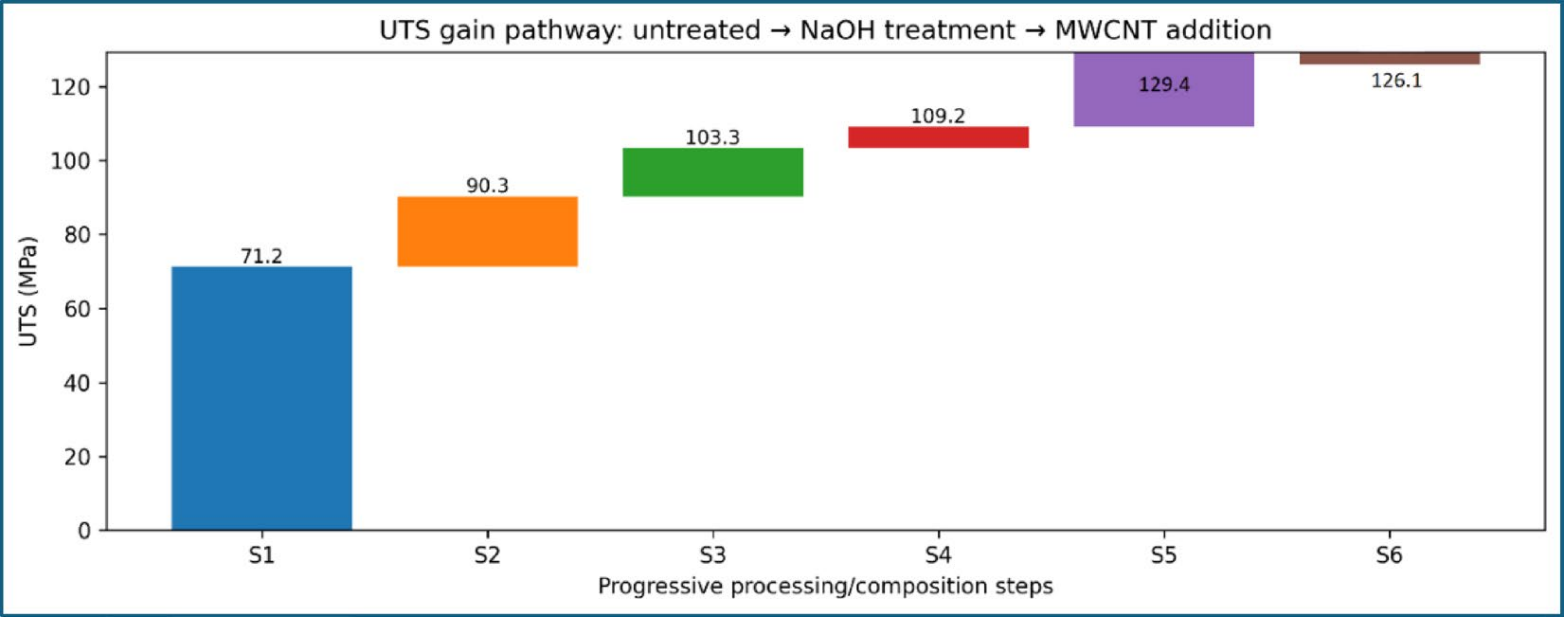
Effect of alkali treatment and MWCNT addition on ultimate tensile strength.

**Fig. 9:**
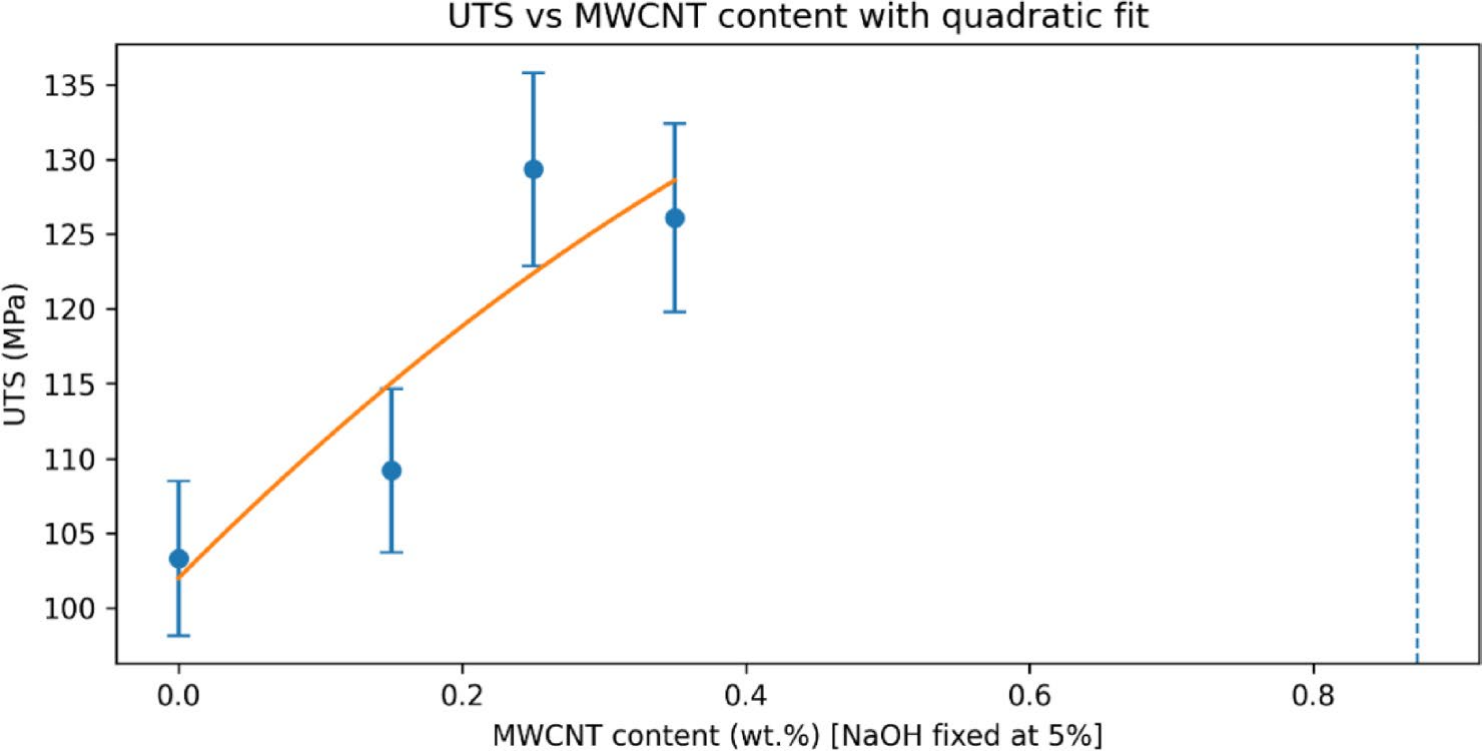
Variation of ultimate tensile strength with MWCNT content at 5 wt% NaOH treatment.

The effect of MWCNT incorporation on the tensile strength of sisal fiber–reinforced epoxy composites was evaluated by varying the CNT content while maintaining the fiber treatment at 5 wt% NaOH, which was identified as the optimal alkali condition in Sect. 4.2. The resulting tensile strength variations are summarized in Fig. 8, while the compositional dependence is further illustrated in Fig. 9; Table 5.

The CNT-free 5 wt% NaOH-treated composite exhibits a UTS of 103.32 ± 5.17 MPa, serving as the reference for assessing the contribution of MWCNT reinforcement. The addition of 0.15 wt% MWCNT leads to a moderate increase in tensile strength to 109.20 ± 5.46 MPa, corresponding to an improvement of approximately 5.7%. This initial enhancement suggests that a low CNT content contributes to matrix stiffening and improved stress transfer without significantly altering the fracture behaviour of the composite. A pronounced strengthening effect is observed at 0.25 wt% MWCNT, where the UTS reaches 129.36 ± 6.47 MPa, representing an increase of approximately 25.2% relative to the CNT-free 5 wt% NaOH-treated composite and 81.6% relative to the untreated composite. As shown in Fig. 9, this composition corresponds to the peak tensile strength within the CNT range investigated. The substantial improvement indicates effective CNT dispersion within the epoxy matrix, enabling efficient crack-bridging and load redistribution mechanisms under tensile loading.

Further increasing the CNT content to 0.35 wt% results in a slight reduction in tensile strength to 126.11 ± 6.31 MPa, although the value remains significantly higher than those of all CNT-free composites. This reduction, visible in Fig. 8, can be attributed to the onset of CNT agglomeration, which introduces localized stress concentrations and limits the reinforcing efficiency of additional CNTs. The relatively small difference between the UTS values at 0.25 wt% and 0.35 wt% CNT, combined with overlapping error margins, indicates diminishing returns rather than abrupt degradation.

### Design-space visualization and processing pathway

**Fig. 10:**
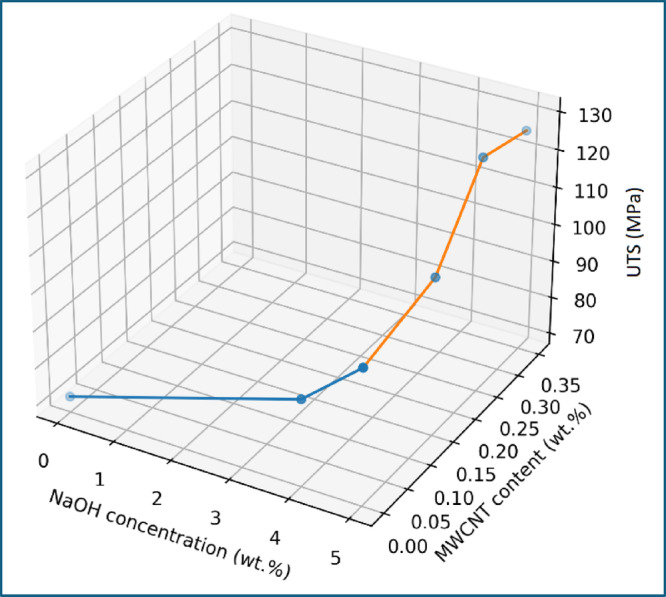
Design-space visualization of alkali treatment and MWCNT reinforcement effects on tensile strength.

To provide a holistic understanding of the combined influence of alkali treatment and MWCNT incorporation on tensile performance, the experimental results were visualized within a three-dimensional design space, as shown in Fig. 10. The axes represent NaOH concentration, MWCNT content, and ultimate tensile strength, enabling simultaneous assessment of processing variables and mechanical response. The data points in Fig. 10 follow a distinct L-shaped processing pathway, reflecting the two-stage experimental strategy adopted in this study. In the first stage, the NaOH concentration varied at zero CNT content, revealing a monotonic increase in tensile strength from untreated to 5 wt% NaOH-treated composites. This segment of the design space highlights the dominant role of fiber surface modification in improving interfacial bonding and load transfer efficiency.

In the second stage, the NaOH concentration was fixed at 5 wt% identified as the optimal alkali treatment and the MWCNT content was varied. This portion of the design path demonstrates a secondary but significant enhancement in tensile strength with CNT addition, culminating in a maximum response at 0.25 wt% MWCNT. The slight decline observed at 0.35 wt% MWCNT further confirms the presence of a practical upper limit for effective nanofiller reinforcement. The separation of the design space into alkali-dominated and CNT-dominated regions underscores the importance of sequential optimization. The visualization clearly indicates that CNT addition is most effective only after establishing a strong fiber–matrix interface through alkali treatment. Direct CNT incorporation without prior interface optimization would therefore be expected to yield suboptimal performance.

### Mathematical modelling and experimental validation

To quantitatively describe the influence of alkali treatment and MWCNT content on tensile strength, regression-based mathematical models were developed using experimentally obtained UTS data. For composites without CNT reinforcement, the effect of alkali treatment was modelled as a second-order polynomial function of NaOH concentration, based on data from untreated, 4 wt% NaOH-treated, and 5 wt% NaOH-treated composites. Although a second-order polynomial regression was fitted, the variation in tensile strength within the investigated NaOH concentration range (0–5 wt%) exhibits near-linear behaviour; therefore, linear trends are emphasized for validation and physical interpretation.

Fitted using S1-S3:

$${\sigma}_{\mathrm{UTS}}=1.661{N}^{2}-1.889N+71.24$$

where $N$ represents the NaOH concentration (wt%).

For CNT-modified composites with NaOH concentration fixed at 5 wt%, the dependence of tensile strength on MWCNT content was modelled using a quadratic relationship fitted to the experimental data:

Fitted using S3-S6:

$${\sigma}_{\mathrm{UTS}}=-54.453{C}^{2}+95.119C+101.987$$

*C* denotes the MWCNT content (wt%).

The quadratic regression yields a mathematical turning point at approximately *C*∗≈0.877 wt% CNT. Since this value lies outside the experimentally investigated range (0–0.35 wt%), it is not interpreted as a physical optimum. The regression is used only to describe the trend within the studied domain, where the highest experimentally observed tensile strength occurred at 0.25 wt% MWCNT.

**Fig. 11:**
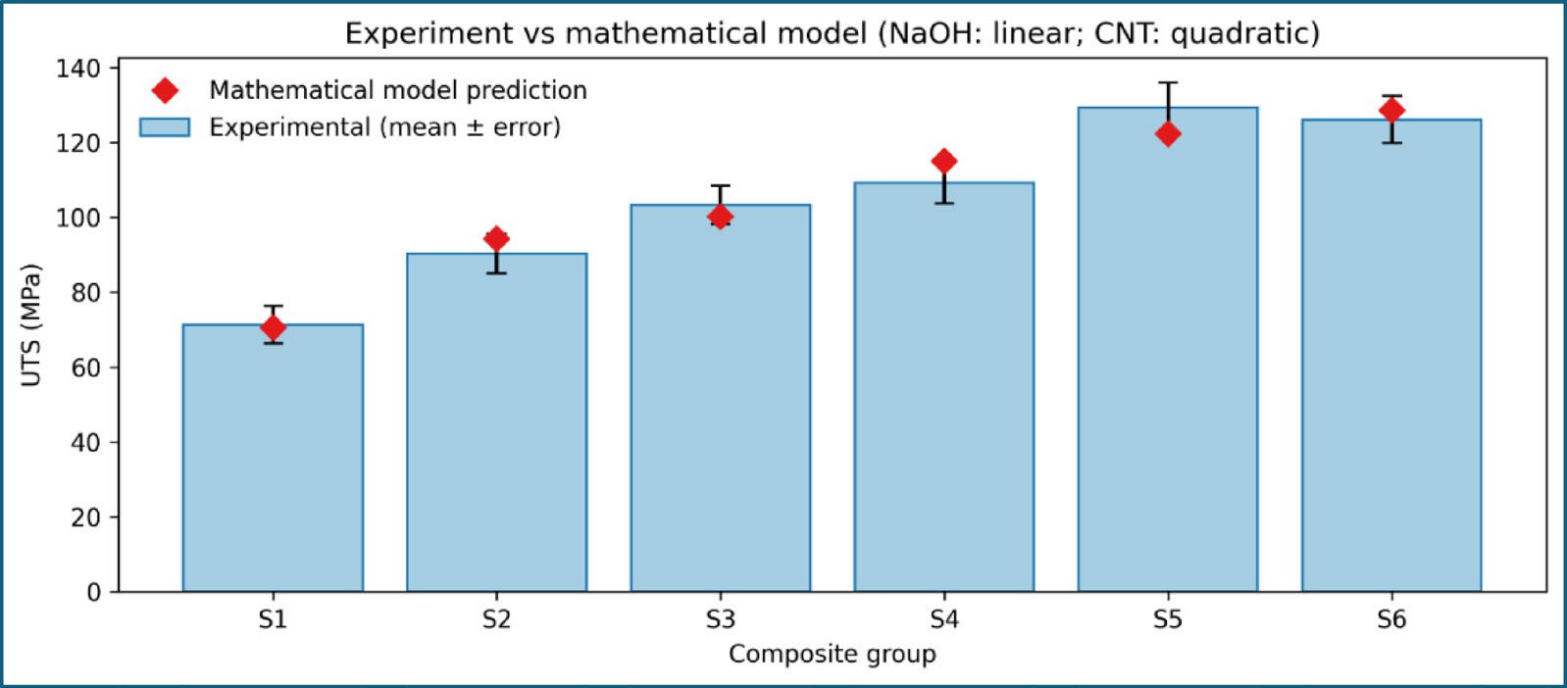
Comparison of experimental and model-predicted ultimate tensile strength.

To validate the physical consistency of the experimentally observed tensile strength trends, mathematical modelling was performed by correlating ultimate tensile strength with alkali treatment level and MWCNT content. The modelling framework was intentionally kept simple and physically interpretable to avoid overfitting and to ensure meaningful comparison with experimental data. The comparison between experimentally measured and model-predicted UTS values is presented in Fig. 11. The model predictions closely follow the experimental trend across all composite formulations, with deviations remaining within the experimental scatter. Importantly, the model does not force perfect agreement with the experimental data, indicating genuine predictive capability rather than curve fitting.

**Fig. 12:**
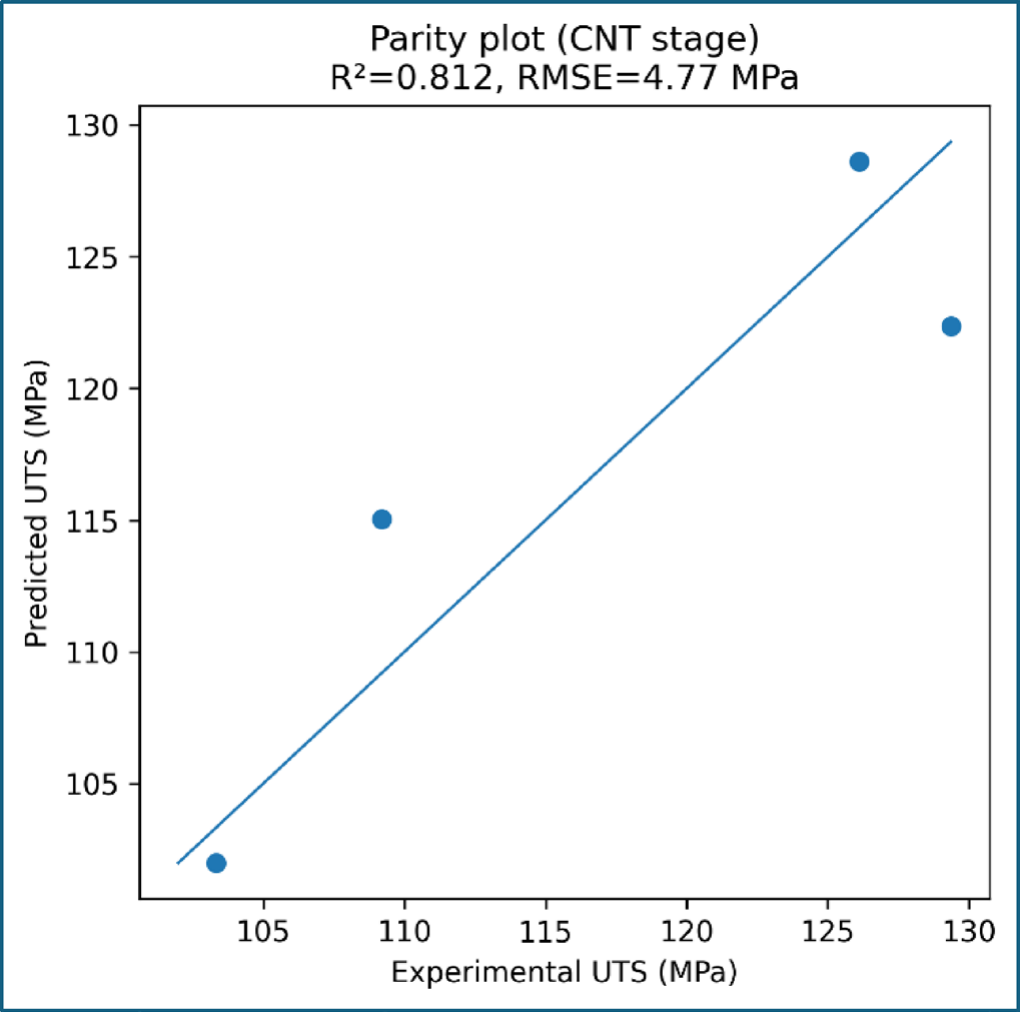
Parity plot comparing experimental and predicted UTS for CNT-modified composites.

**Fig. 13:**
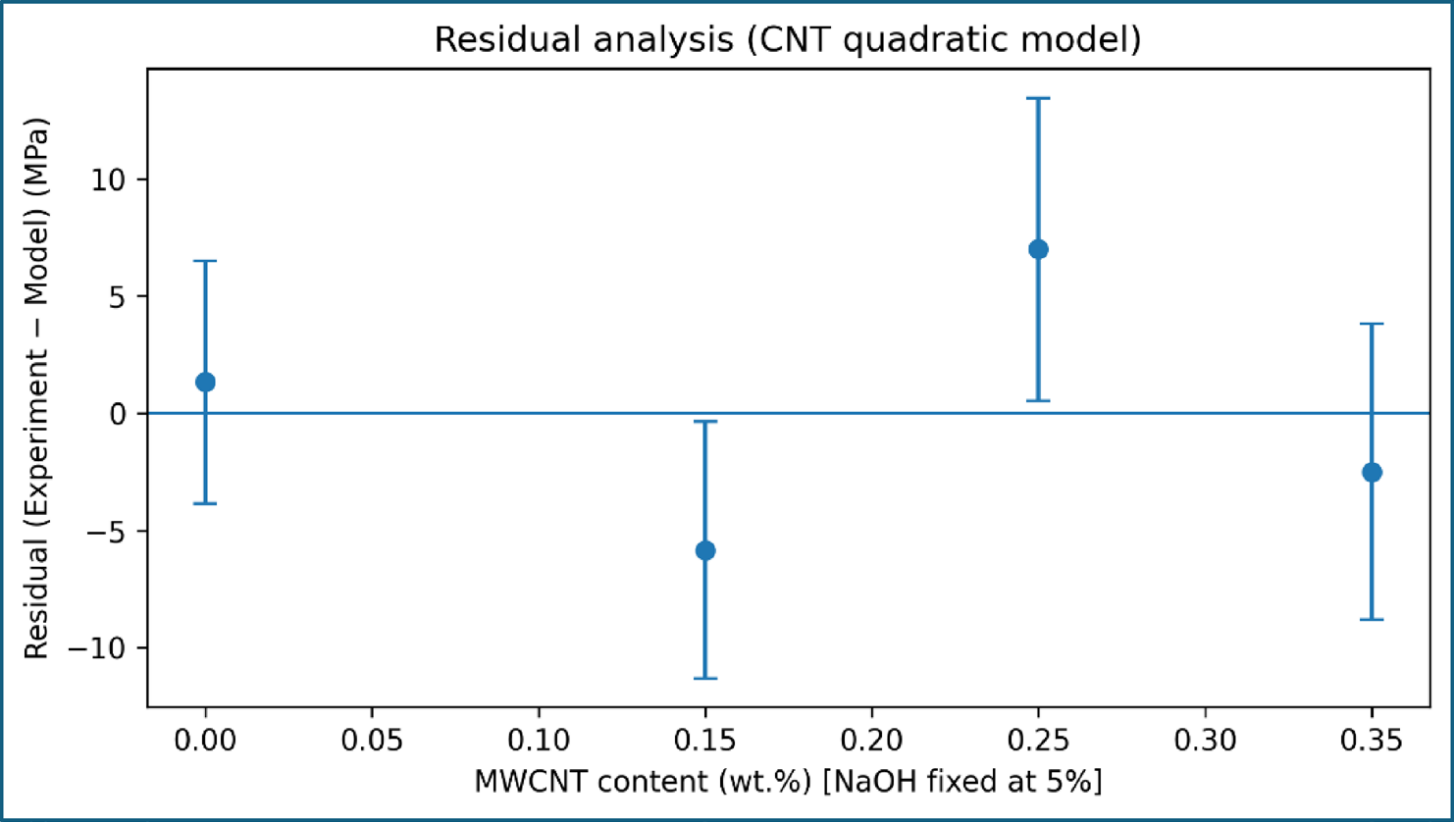
Residual analysis of model predictions for CNT-modified composites.

The predictive accuracy of the CNT-stage model is further assessed using the parity plot shown in Fig. 12, where experimental and predicted UTS values cluster near the 1:1 reference line. The limited dispersion around the parity line confirms that the quadratic model adequately captures the non-linear dependence of tensile strength on MWCNT content within the investigated range. Residual analysis, presented in Fig. 13, reveals no systematic bias in the model predictions. The residuals fluctuate around zero across the CNT content range, indicating that the deviations arise primarily from experimental variability rather than deficiencies in the modelling approach. The magnitude of the residuals remains comparable to the reported experimental error, reinforcing the reliability of the model.

**Fig. 14:**
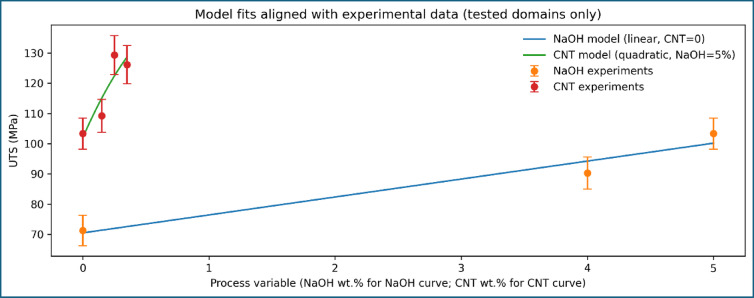
Regression models describing NaOH and MWCNT effects on UTS.

**Fig. 15:**
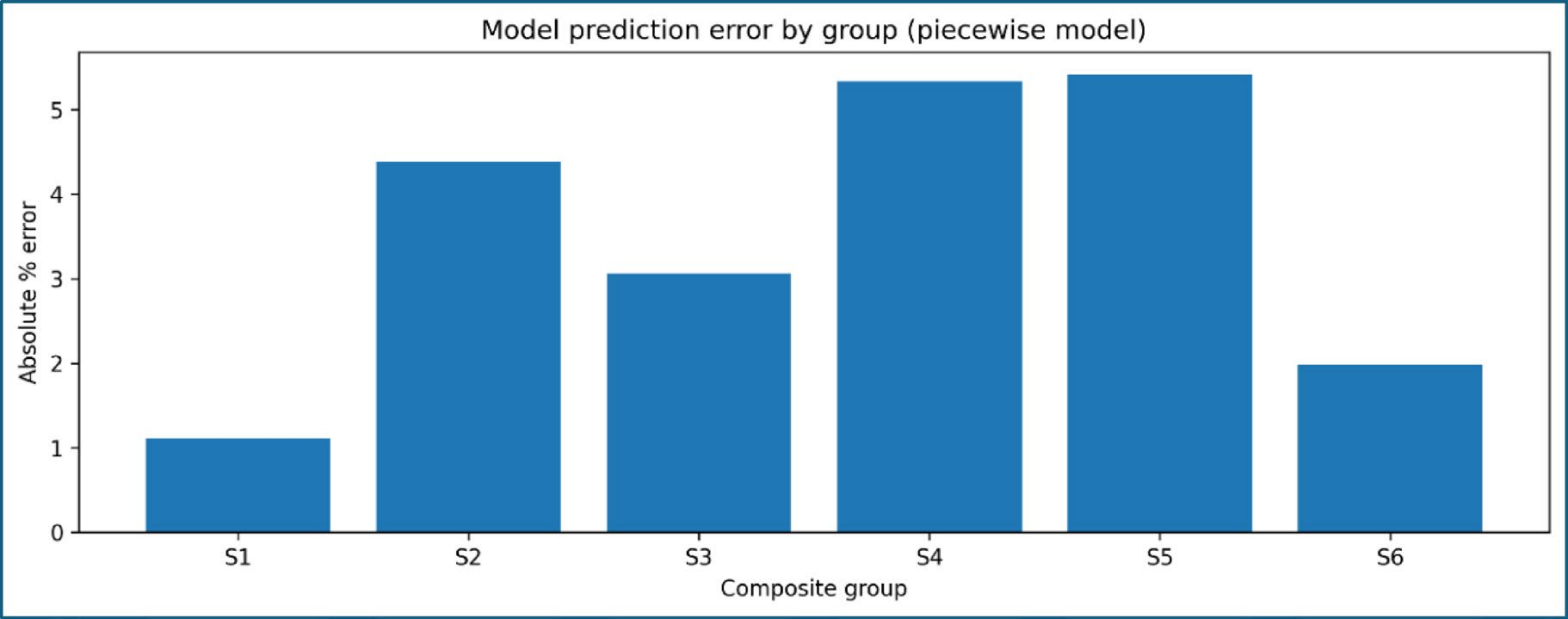
Absolute percentage error between experimental and model-predicted UTS.

The suitability of the selected modelling forms is illustrated in Fig. 14, which overlays the experimental data with the corresponding regression curves. A linear relationship effectively describes the influence of alkali treatment at zero CNT content, reflecting progressive improvement in fiber–matrix interfacial bonding. In contrast, a quadratic relationship is required to capture the CNT-induced strengthening behaviour, accounting for both reinforcement efficiency at low CNT contents and the onset of saturation at higher loadings. The overall modelling uncertainty is quantified in Fig. 15; Table 6 shows the toughness, which present the absolute percentage error for each composite formulation. The errors remain within acceptable limits and are consistently lower than the experimental uncertainty associated with tensile testing. This agreement confirms that the proposed modelling framework accurately represents the experimental trends without relying on extrapolation beyond the tested domain.

**Table 6:** Toughness values derived from stress–strain curves.

Sample ID	Composite description	Toughness (MJ·m⁻³)
S1	Untreated sisal/epoxy	1.95 ± 0.15
S2	4 wt% NaOH-treated sisal/epoxy	2.35 ± 0.18
S3	5 wt% NaOH-treated sisal/epoxy	2.80 ± 0.20
S4	5 wt% NaOH + 0.15 wt% MWCNT	1.10 ± 0.09
S5	5 wt% NaOH + 0.25 wt% MWCNT	1.25 ± 0.10
S6	5 wt% NaOH + 0.35 wt% MWCNT	1.40 ± 0.12

**Fig. 16:**
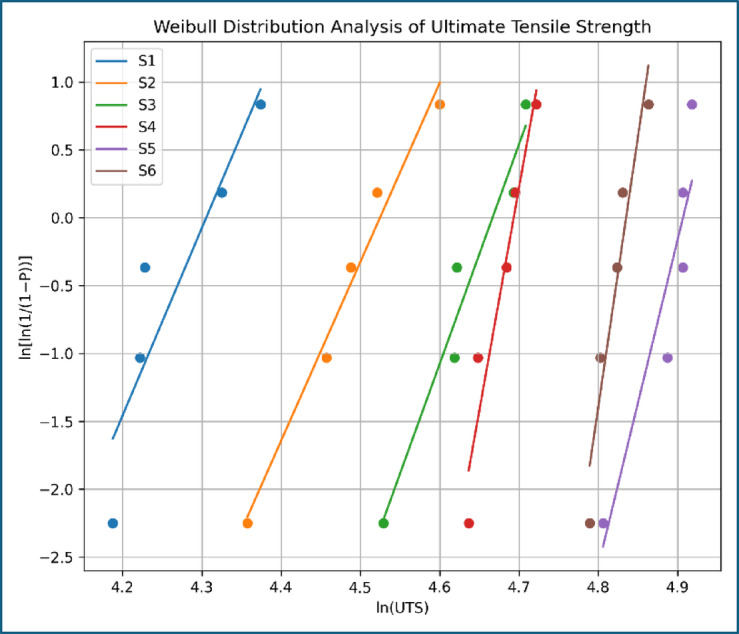
Weibull distribution analysis of ultimate tensile strength.

Figure 16 presents the Weibull distribution analysis of ultimate tensile strength for composites S1–S6, providing insight into the statistical reliability of the measured tensile response. The untreated composite (S1) exhibits the lowest Weibull slope, indicating high scatter due to weak fiber–matrix bonding and interfacial failure. Progressive steepening of the Weibull plots for S2 and S3 reflects reduced variability resulting from alkali-induced interface improvement. The CNT-modified composites further show enhanced reliability, with S5 (0.25 wt% MWCNT) exhibiting the highest Weibull modulus, consistent with its superior tensile strength, elastic modulus, and cohesive fracture morphology observed in SEM analysis. A slight reduction in slope for S6 confirms increased scatter due to CNT agglomeration, supporting the observed saturation and marginal decline in mechanical performance at higher CNT loading. Weibull analysis was performed to compare strength variability among formulations. The results are interpreted in a comparative sense rather than for predictive reliability modeling and the parameters are shown in Table 7.While larger sample sizes improve statistical confidence in Weibull parameters, the present dataset is sufficient to reveal relative differences among formulations.

**Table 7:** Weibull parameters.

Sample	Weibull modulus m	Characteristic strength σ₀ (MPa)	*R*²
S1	8.2	74.1	0.96
S2	9.5	93.6	0.97
S3	10.8	107.5	0.97
S4	11.2	112.6	0.96
S5	12.4	133.1	0.98
S6	10.6	129.4	0.95

###  Fracture surface morphology and interface-controlled failure mechanisms

**Fig. 17:**
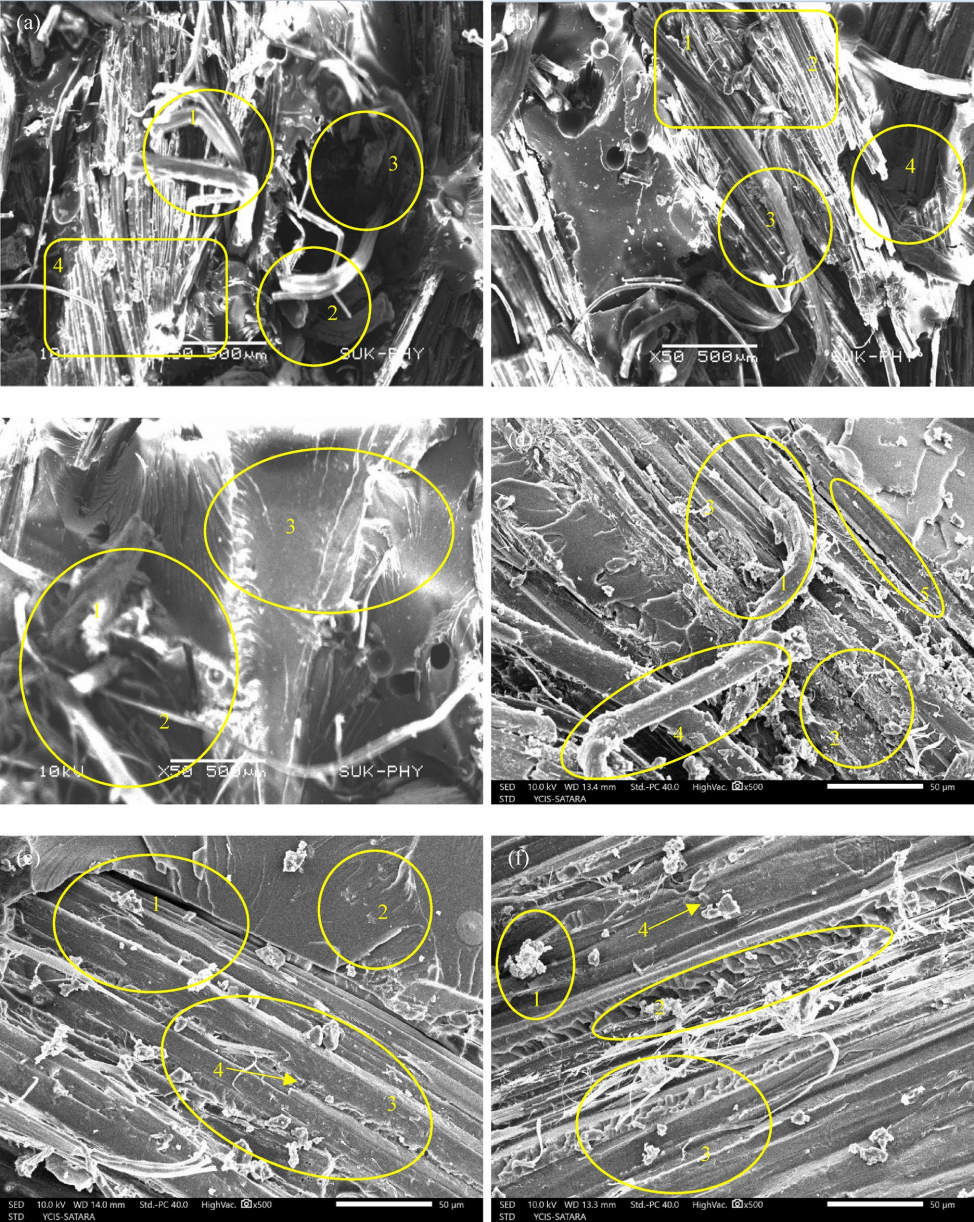
SEM fractographs (**a**) S1 (**b**) S2 (**c**) S3 (**d**) S4 (**e**) S5 (**f**) S6.

Table 8 links the numbered SEM regions in Fig. 17(a–f) to the tensile response of the six laminates by showing how interface integrity and matrix morphology control stiffness (E), strength (UTS), and ductility (εf). For S1 Fig. 17(a), the fracture surface is dominated by long, clean fiber pull-out, smooth fiber walls, and clear interfacial gaps, which indicates early interfacial slip and inefficient load transfer; this directly explains the lowest stiffness and strength (E = 4.8 GPa; UTS = 71.24 MPa) together with the highest ductility (εf = 2.95%), as deformation is accommodated by progressive debonding and pull-out rather than rapid catastrophic fracture.

In S2 Fig. 17(b), roughened fibers with partial resin adherence and reduced pull-out lengths confirm improved mechanical interlocking after 4 wt% NaOH treatment. This morphology supports the observed increase in stiffness and strength (E = 6.2 GPa; UTS = 90.26 MPa) while maintaining a similar strain at break (εf = 2.9%), indicating that the interface is strengthened without severely restricting deformation mechanisms. For S3 Fig. 17(c), the strongest fibrillation, tight matrix wrapping, and frequent fiber fracture (rather than pull-out) indicate that the interface has become sufficiently strong to transfer load into the fibers effectively; consequently, both modulus and UTS increase further (E = 6.9 GPa; UTS = 103.32 MPa). The slightly higher εf (3.05%) is consistent with a more stable damage evolution where the interface resists debonding and the composite sustains deformation through distributed microcracking and fiber breakage instead of premature interfacial failure.

**Table 8:** SEM fractographic features identified at marked regions for composites.

ID	Key SEM features	Dominant fracture/interface characteristics
S1	(1) Long, clean fiber pull-out (2) Smooth fiber surfaces (3) Clear gaps between fiber and matrix (4) Relatively flat matrix regions	Weak fiber–matrix adhesion leading to interfacial debonding and extensive fiber pull-out
S2	(1) Roughened fiber surfaces (2) Partial matrix adhesion on fibers (3) Shorter pull-out lengths (4) Fibrillation of fiber walls	Improved interfacial bonding due to alkali treatment, but still governed by mixed pull-out and partial debonding
S3	(1) Strongest fibrillation (2) Fibers often fractured rather than pulled out (3) Matrix tightly wrapped around fibers	Highly efficient load transfer with fiber fracture dominated failure indicating optimal interface strength
S4	(1) Slight matrix roughening (2) CNT -rich regions (3) Interface improved (4) But still some pull-out (5) Crack deflection limited	Initial matrix toughening due to CNT addition with moderate crack deflection and improved interface continuity
S5	(1) Highly cohesive fracture surface (2) Matrix uniformly rough (fine granular texture) (3) Fibers almost fully embedded (4) Minimal voids Crack bridging and deflection evident	Optimal CNT dispersion leading to maximum energy absorption, effective crack bridging, and highest tensile strength
S6	(1) Presence of larger agglomerates (2) Voids around clusters (3) Local debonding despite overall good interface (4) Some cracks emanating from cluster regions	Over-reinforcement effects where CNT agglomeration induces stress concentration and premature local failure

Moving to CNT-modified systems, the SEM of S4 Fig. 17(d) shows a distinctly roughened matrix with sparse CNT-rich clusters and limited crack deflection, indicating matrix stiffening with only partial toughening. This aligns with the increase in modulus (E = 7.4 GPa) and a modest rise in UTS (109.2 MPa), but the fracture surface features also imply a transition toward more brittle failure: the reduced pull-out and more cohesive matrix regions coincide with the sharp drop in strain at break (εf = 1.2%). In S5 Fig. 17(e), the most cohesive and uniformly rough fracture morphology minimal voids, fibers almost fully embedded, and clear evidence of crack deflection/bridging supports simultaneous maximization of stiffness and strength (E = 8.1 GPa; UTS = 129.36 MPa). The low εf (1.15%) indicates that although the composite is stronger and stiffer, failure occurs after a shorter deformation window, consistent with a stiffened CNT-modified matrix and a strong interface that suppresses pull-out-based energy dissipation.

For S6 Fig. 17(f), SEM features characteristic of overloading larger agglomerates, voids around clusters, and localized debonding with cracks emanating from these regions explain why stiffness and strength no longer increase (E decreases slightly to 7.9 GPa; UTS reduces marginally to 126.11 MPa). At the same time, εf increases to 1.5% relative to S4–S5, which is consistent with non-uniform stress redistribution: local defects promote early microcrack initiation but can also allow limited deformation through crack coalescence and local debonding before final fracture. Overall, the SEM evidence confirms that the NaOH-driven interface improvement (S1→S3) enhances both stiffness and strength while retaining ductility, whereas CNT addition (S3→S5) primarily increases modulus and UTS through matrix stiffening and crack-path tortuosity, but reduces εf due to a more constrained, cohesive failure mode; excessive CNT content (S6) introduces agglomeration defects that limit further gains and partially restore ductility by enabling localized damage mechanisms.

**Fig. 18:**
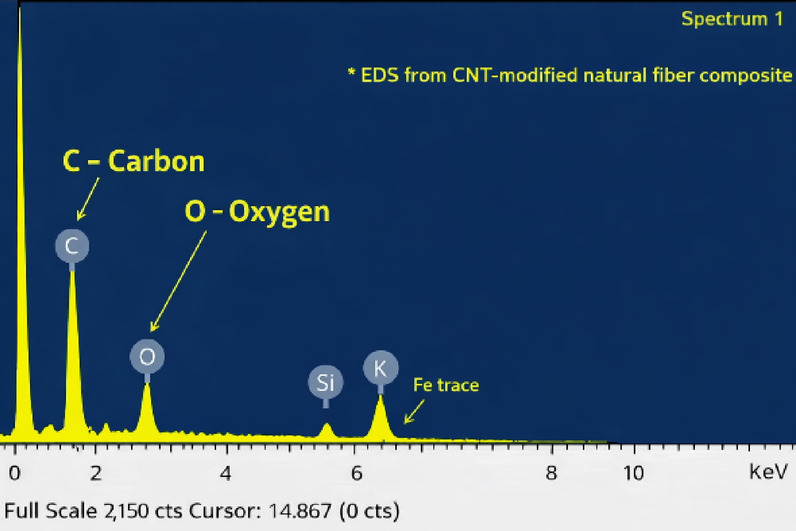
EDS spectra at the fractured surface.

EDS analysis (Fig. 18) showed dominant carbon and oxygen peaks consistent with the bio-epoxy, lignocellulosic fibers, and CNT-modified matrix, confirming composite composition and reinforcement incorporation. As EDS provides elemental rather than molecular information, the observed cohesive fracture is interpreted in terms of improved interfacial interaction and mechanical interlocking rather than direct chemical bonding.

## Conclusion

This study demonstrates that a sequential reinforcement strategy combining alkali-treated sisal fibers and low-content MWCNT-modified bio-epoxy matrices provides a highly effective route for improving the tensile performance and reliability of natural fiber composites. Alkali treatment alone increased the ultimate tensile strength from 71.24 MPa for untreated composites to 103.32 MPa at 5 wt% NaOH, corresponding to an improvement of approximately 45%, while the elastic modulus increased from 4.8 GPa to 6.9 GPa, representing a 44% enhancement due to improved fiber–matrix interfacial bonding.

Subsequent matrix modification using MWCNTs further enhanced mechanical performance. Incorporation of 0.25 wt% MWCNT into the 5 wt% NaOH-treated composite increased the ultimate tensile strength to 129.36 MPa, yielding an additional 25% improvement over the CNT-free 5 wt% NaOH-treated composite and an overall 81% increase compared to the untreated system. The elastic modulus reached a maximum of 8.1 GPa, corresponding to a 17% increase over the alkali-treated composite and a 69% increase relative to the untreated composite. These gains were achieved with a reduction in strain at break from 3.05% to 1.15%, indicating a transition from ductile interfacial failure to a stiffer, more cohesive fracture mechanism.

Mathematical modelling captured the near-linear strengthening response associated with alkali treatment and the non-linear saturation behavior associated with CNT addition, with predicted trends remaining within experimental scatter. Weibull statistical analysis revealed a progressive reduction in tensile strength variability for alkali-treated and optimally CNT-modified composites, confirming improved mechanical reliability. Fractographic analysis provided direct microstructural validation of these trends, revealing a systematic transition from fiber pull-out–dominated failure in untreated composites to cohesive fracture and crack-bridging mechanisms at optimal CNT loading, while excessive CNT addition (0.35 wt%) introduced agglomeration-induced defects that resulted in a slight ~ 2.5% reduction in tensile strength compared to the optimum.

Overall, the results establish a quantitative structure property reliability framework for designing high-performance natural fiber composites using renewable resources and minimal nanofiller content. The demonstrated improvements in strength, stiffness, and reliability directly support sustainable materials innovation (SDG 9), responsible material utilization through reinforcement optimization (SDG 12), and climate-conscious lightweight composite development aimed at reducing environmental impact across the material lifecycle (SDG 13).

## Data Availability

All data supporting the findings of this study are available within the paper and its Supplementary Information.
